# Detection, virulence and genetic diversity of *Fusarium* species infecting tomato in Northern Pakistan

**DOI:** 10.1371/journal.pone.0203613

**Published:** 2018-09-20

**Authors:** Asma Akbar, Shaukat Hussain, Kaleem Ullah, Muhammad Fahim, Gul Shad Ali

**Affiliations:** 1 Mid-Florida Research and Education Center, Department of Plant Pathology, University of Florida, Institute of Food and Agricultural Sciences, Apopka, FL, United States of America; 2 Khyber-Pakhtumkhwa Agriculture University, Peshawar, Pakistan; Oklahoma State University, UNITED STATES

## Abstract

In addition to the well-known *Fusarium oxysporum* f.sp. *lycopersici*, several other *Fusarium* species are known to cause extensive worldwide crop losses in tomatoes. Prevalence and identities of *Fusarium* species infecting tomatoes in Northwest Pakistan is currently not known. In this study, we surveyed and characterized *Fusarium* species associated with symptomatic tomatoes in Northwest Pakistan using morphological and molecular analyses. Pathogenicity tests revealed varying degrees of virulence with some *Fusarium* sp. causing severe disease symptoms whereas others displaying mild symptoms. Molecular identification based on Internal Transcribed Spacer (ITS) region and *TEF-1α* gene sequencing classified all isolates into four major species with a majority (68.9%) belonging to *Fusarium incarnatum-equiseti species complex* (FIESC), followed by *F*. *graminearum* (20.7%), *F*. *acuminatum* (6.8%), and *F*. *solani* (6.8%). ISSR analyses revealed substantial genetic variability among all the *Fusarium* population infecting tomatoes. Genetic distance between populations from the central region and the type strain *F*.*o*. f.sp. *lycopersici* from Florida was the highest (0.3662), whereas between the south and central region was the lowest (0.0298), which showed that genetic exchange is negatively effected by distance. High genetic variability suggests that these *Fusarium* species have the potential to become a major production constraint for tomato growers. Findings in this report would greatly facilitate identification of *Fusarium* species in developing countries and would provide groundwork for devising and implementing disease management measures for minimizing losses caused by *Fusarium* species in tomatoes.

## Introduction

Tomato (*Solanum lycopersicum*), a member of family Solanaceae, is considered one of the world’s most important and popular vegetables [1]. It is ranked sixth among the most popular vegetables by the Food and Agriculture Organization, in terms of total annual production in the world. Globally 159 million tons of fresh tomatoes are produced annually, of which about a quarter are grown for processing, making tomato a leading vegetable for each of the nine largest agricultural producing countries—China, India, USA, Turkey, Egypt, Iran, Italy, Brazil, and Spain—who account for more than 74% of the world’s annual production. In Pakistan, tomatoes have a significant economic value. Consumption and demand for tomatoes are increasing steadily due to increase in population, and it is available at a lower price in comparison to other vegetables with higher nutritional values. It is consumed as ketchup, sauces, a fresh vegetable, and more in many home-cooked and restaurant-served meals. Furthermore it is available year-round due to its seasonal production in different locations worldwide [2]. Pakistan exported 9832 tons of tomatoes from 2009–2010 valued at 77 million rupees. During this time period, the production area increased from 27.9 to 50 thousand hectares, with an increase in yield from 268.8 to 476.8 thousand tons. Based on a ten years average, the present Pakistani national yield of tomatoes is 10 tons/ha, which is quite low relative to other tomato producing counties. In Baluchistan, 18 thousand hectares is cultivated, placing it on the top among all Pakistani provinces, while Khyber Pakhtunkhwa (KP) and Sindh cultivate 15.6 and 10.7 thousand hectares, respectively. Europe and America are the major sources of commercially grown cultivars that are introduced to the country [3].

There are extensive worldwide crop losses in tomato production due to multiple *Fusarium* species [4]. The pathogen resides in the soil, infecting plants through their roots and crown. Different species of Fusarium are associated with wilt of tomatoes such as *Fusarium verticillioides*, *F*. *oxysporum*, and *F*. *equiseti* [5]. They can infect tomatoes through both roots and crown area at any growth stage. Some species, such as *Fusarium oxysporum*, infect vascular bundles, causing infected plants show an early wilting syndrome due to stress [6, 7]. Fusarium wilt is more common in acidic sandy soils. The wilt pathogen can reside in infested soil for a period of up to ten years. Soil with very warm (34°C) or cool (17-20°C) temperatures slow down wilt development [8].

*Fusarium* species’ population in Khyber Pakhtunkhwa has not been studied previously. It is not clear whether *Fusarium* species currently infecting tomatoes in this region represent single or multiple species and whether they are monomorphic or polymorphic, both of which have serious implications for disease management.

An internal transcribed spacer (ITS) is a molecular marker widely used for the identification, molecular ecology, and phylogenetic analysis in eukaryotes including fungi [9, 10]. Earlier studies focused on phylogeny of *Fusarium* have also used ITS, however, with the discovery of new closely related species in a given *Fusarium* species complex, ITS has been found not suitable in resolving closely related species [11]. In contrast to ITS, several conserved genes, such as actin, translation elongation factor1- α (*TEF-1α*), *α*-tubulin, β-tubulin (TUB), and RNA polymerase II subunits 1 and 2 (RPB1 and RPB2), have recently been used for phylogenetic analyses of Fusarium species [12]. All these genes providerelatively similar phylogenetic results and usually only one of them is recommended for providing sufficient genetic resolution [11, 13]. Many recent studies have reported using the *TEF-1α* for the identification of *Fusarium* species and for discripmating to the subspecies levels [11, 14–16].

Comprehensive understanding of pathogen populations and diversity at the species level is important, as high genetic diversity indicates a rapid change in genetic structures. This, in turn, shows the development of more virulent species and strains in response to management practices, changed environments, and increased biological fitness of these species [17]. Screening the pathogen population, therefore, helps us in studies aimed at reliably detecting variations in the population structure of the pathogen, which in turn enable us to understand population biology of the pathogen [18]. Many studies reported that molecular techniques using different markers could detect genetic diversity within and among populations to a certain extent, including pathogenicity variation, geographical, and host differences [19]. The development of DNA markers such as ISSR (Inter Simple Sequence Repeats) to examine population dynamics of the pathogen helped us to quantify diversity much more efficiently by providing levels of precision not previously available [20–22]. ISSR are dominant genetic markers [23] generated by amplification of genomic DNA using single primer through polymerase chain reaction (PCR), provided the primer binding sites are located within the amplifiable range that is inversely oriented simple sequence repeats (SSR). Researchers frequently use ISSR markers for genetic diversity within the species, characterization of germplasm, cultivar identification, and phylogenetic studies at the species level [24, 25]. Due to its high reproducibility and cost effectiveness, the ISSR technique is preferred to the Random Amplified Polymorphic DNA (RAPD) and Amplified Fragment Length Polymorphism (AFLP) techniques [26]. Furthermore, individuals of the same species show little to no difference between ISSR bands, but closely related species and subspecies yield specific ISSR patterns that can be used for phylogenetic relatedness [27].

This study was carried out to report the incidence and severity of *Fusarium* wilt of tomato and to identify and study the genetic diversity of *Fusarium* species associated with wilt of tomato in Khyber-Pakhtunkhwa (KP) using ITS and ISSR markers.

## Materials and methods

### Survey, sample collection and disease prevalence

Multiple surveys were conducted in major tomato growing areas of the Khyber-Pakhtunkhwa province in Northwest Pakistan. Seven districts–Swat, Malakand, Mansehra, Charsadda, Swabi, Peshawar and Bannu–where there was a reported occurrence of *Fusarium* wilt of tomato were selected for the study. Geographical coordinates of the surveyed locations are provided in Table 1. In each district, five areas per district and four fields per area were surveyed. From each area, two representative farmers were selected, who where then interviewed based on a structured questionnaire to determine tomato cultivars grown, incidences of *Fusarium* wilt, and methods used by the farmers to control the disease. In addition to farmers, an agriculture officer was also interviewed in each area. The survey pattern was random, with an x-shaped path covering the entire field, including both healthy and infected samples. The incidence of the disease was calculated as follows: Disease incidence (%) = (*n*/N) × 10, where, *n* = number of plants infected and N = total number of plants examined. The severity of the disease was determined using the following 0–4 indexing scale with slight modification [28]. Disease Severity Scale: 0 = healthy plants; 1 = initiation of wilt symptoms; 2 = wilting, yellowing and browning up to 50%; 3 = plants wilted, yellow brown discoloration more pronounced, and entire plants started dying (75%); 4 = complete drying and dying of the entire plant due to wilt. Percent severity of disease was calculated as follows:

Diseaseseverity(%)=∑[(n×V)/(5×N)]×100

Where *n* = number of plants infected, *V* = numerical value of infected plants, *N* = total number of plants examined and 5 = the highest infection category.

**Table 1:** Geographical distribution, pathogenicity, virulence and morphological characteristics of *Fusarium* species in Khyber Pakhtunkhwa.

District	Location	ID	Patho-genesis	Vir^a^	Culture color Upper/lower	MSL of Macro-conidia^b^	MSL of Micro-conidia^b^	Macro-conidia septation	Chlamydo-spores	Morpho-logical identification	ITS	*TEF-1α*	ISSR^c^
Swat	Shingardar 34.692332 N 72.248410 E	Pak-1	+	HV	Whitish Brown/Brown	37.7±7.5	9.7±1.6	3–5	-	*F*. *equiseti*	*F*. *equiseti*	FIESC	3
	Ghalegay 34.694713 N 72.263923 E	Pak-2	+	LV	Whitish Brown/Brown	35.2±2.4	Nd^d^	3–5	+	*F*. *equiseti*	*F*. *equiseti*	FIESC	3
	Shamozai 34.684289 N 72.124904 E	Pak-3	+	LV	Whitish Brown/Brown	44.0±3.8	Nd	3–5	+	*F*. *equiseti*	*F*. *equiseti*	FIESC	3
	Takhtaband 34.781572 N 72.326408 E	Pak-4	+	MV	White/Whitish Brown	Nd	Nd	3–5	+	*F*. *equiseti*	*F*. *equiseti*	FIESC	3
Mansehra	Bajna 34.450222 N 73.249998 E	Pak-5	+	LV	Whitish Brown/ reddish Brown	31.2±1.2	Nd	3–5	+	*F*. *equiseti*	*F*. *equiseti*	FIESC	3
	Khaki 34.406899 N 73.132410 E	Pak-6	+	HV	White/ orange brown	33.0±3.9	Nd	3–5	+	*F*. *equiseti*	*F*. *equiseti*	FIESC	3
	Panodehri 34.365136 N 73.196643 E	Pak-7	-	-	Yellowish white/Yellow	30.6±1.6	Nd	3–6	+	UD	*F*. *acuminatum*	*F*. *acuminatum*	2
	Bafamera 34.442164 N 73.221451 E	Pak-8	-	-	Yellowish white/Yellow	28.6±1.7	Nd	3–6	+	UD	*F*. *acuminatum*	*F*. *acuminatum*	2
	Shinkiari 34.456839 N 73.268990 E	Pak-9	+	LV	Whitish Brown/Brown	35.4±4.8	Nd	3–5	-	*F*. *equiseti*	*F*. *equiseti*	FIESC	3
Charsadda	Harichand 34.384007 N 71.800828 E	Pak-10	-	MV	White/Pink-orange	32.0±2.8	Nd	4–6	-	*F*. *graminearum*	*F*. *graminearum*	*F*. *graminearum*	1
	Mandani 34.342474 N 71.788651 E	Pak-11	+	MV	Whitish brown/brown	25.7±3.0	Nd	3–5	-	*F*. *equiseti*	*F*. *equiseti*	FIESC	3
	Prang Ghar 34.408473 N 71.638629 E	Pak-12	+	LV	White/Brown	32.5±3.8	Nd	3–5	-	*F*. *equiseti*	*F*. *equiseti*	FIESC	3
	Baramderai 34.361107 N 71.606420 E	Pak-13	-	-	Olive green/ Light Brown	29.9±4.4	Nd	4	-	Unidentified	*F*. *verticiliodes*	*F*. *graminearum*	3
	Shakoor 34.386749 N 71.737914 E	Pak-14	-	-	White/Brown	38.6±2.1	Nd	4–6	+	*F*. *graminearum*	*F*. *graminearum*	*F*. *graminearum*	1
Peshawar	Malakandair 34.009530 N 71.466391 E	Pak-15	+	MV	White/Whitish Brown	39.1±1.8	Nd	3–5	+	*F*. *equiseti*	*F*. *equiseti*	FIESC	3
	Pakhaghulam 34.037106 N 71.612222 E	Pak-16	+	LV	Whitish Brown/Brown	56.7±3.5	Nd	3–5	-	*F*. *equiseti*	*F*. *equiseti*	FIESC	3
	Gari Baloch 33.978758 N 71.572019 E	Pak-17	+	LV	Whitish Brown/Brown	30.6±2.8	Nd	3–5	-	*F*. *equiseti*	*F*. *equiseti*	FIESC	3
	Palosai 34.024437 N 71.478464 E	Pak-18	+	LV	Whitish Brown/Brown	32.1±3.7	Nd	3–5	-	*F*. *equiseti*	*F*. *equiseti*	FIESC	3
	Taranab 34.012188 N 71.707446 E	Pak-19	-	-	White/ Brown	34.5±4.0	Nd	4–6	-	*F*. *graminearum*	*F*. *graminearum*	*F*. *graminearum*	1
Malakand	Dargai 34.506912 N 71.905709 E	Pak-20	+	MV	Whitish Brown/Creamy	33.6±6.5	Nd	3–5	+	*F*. *equiseti*	*F*. *equiseti*	FIESC	3
	Sakhakot 34.460950 N 71.915725 E	Pak-21	+	MV	Pale to brown/creamy	29.0±5.3	4.3±1.0	3–7	-	*F*. *solani*	*F*. *solani*	*F*. *solani*	3
	Thana 34.632505 N 72.062101 E	Pak-22	+	MV	White/Brown	34.8±3.0	Nd	3–5	+	*F*. *equiseti*	*F*. *equiseti*	FIESC	3
	Chakdara 34.663531 N 72.025960 E	Pak-23	+	MV	Brown/Reddish Brown	29.9±4.2	Nd	3–5	-	*F*. *equiseti*	*F*. *equiseti*	FIESC	3
	Batkhela 34.613545 N 71.926500 E	Pak-24	+	MV	Whitish Brown/Brown	32.2±2.5	Nd	3–5	-	*F*. *equiseti*	*F*. *equiseti*	FIESC	3
Swabi	Maini 34.119097 N 72.605246 E	Pak-25	+	MV	Whitish Brown/Brown	36.0±4.2	Nd	3–5	-	*F*. *equiseti*	*F*. *equiseti*	FIESC	3
Bannu	Sarainurang 32.828164 N 70.778491 E	Pak-26	-	-	White/Dark Pink	40.3±5.4	Nd	3–6	-	Unidentified	*F*. *acuminatum*	*F*. *graminearum*	2
	Kakki 34.407864 N 73.131885 E	Pak-27	-	-	Whitish Brown/Reddish brown	36±4.2	Nd	4–6	-	*F*. *graminearum*	*F*. *graminearum*	*F*. *graminearum*	1
	Ghoriwala 32.904753 N 70.730865 E	Pak-28	+	MV	White/Dark Brown	34± 4.3	Nd	3–5	+	*F*. *equiseti*	*F*. *equiseti*	FIESC	3
	Khojar Ismail Khani 32.952960 N 70.650271 E	Pak-29	+	MV	Pale to brown/creamy	33.1±2.5	5.1±1.5	3–7	-	*F*. *solani*	*F*. *solani*	*F*. *solani*	3
Florida		Fl-15			White/Brown	23.3±3.3	4.9±1.7	3–5	-	*F*. *oxysporum*	*F*. *oxysporum*	*F*. *oxysporum*	

^a^ Vir = virulence levels; HV, highly virulent; MV, moderately virulent; LV, low virulent;–, non-virulent

^b^MSL = mean spore length of macro and microconidia

^c^ISSR = 1,2,3 refers to different group (Clades) of the isolates based on ISSR analysis

^d^Nd = Not determined

### Collection of tomato plants and soil samples, isolation and maintenance of *Fusarium* species

Symptomatic and asymptomatic tomato tissues were collected during survey from each representative area in Khyber-Pakhtunkhwa (KP) province. All samples were placed in labelled brown paper bags and transported to the Department of Plant Pathology at the University of Agriculture, Peshawar, Pakistan and stored at 4°C for further processing. From symptomatic and asymptomatic collected samples, leaves and secondary roots were trimmed off, leaving only the main stem, hypocotyls, and the main root. The stems of a diseased sample with obvious symptoms and stems of visually healthy asymptomatic samples were excised laterally to expose the xylem tissues just beneath the epidermis. The exposed stems were then cut into small 1 cm long pieces, surface sterilized by soaking in 10% sodium hypochlorite solution (NaOCl) for 1 minute, washed with sterile distilled water thrice and blot dried between the folds of paper towels. The stems were then cut into 2 to 4 mm thick wedges and placed on potato dextrose agar (PDA) plates amended with 30 mg/L streptomycin to inhibit bacterial growth. The plates were then incubated at 25°C for seven days to allow for the development of mycelial growth of *Fusarium* species. A pure culture of each isolates was stored on PDA slants at 4°C.

### Pathogenicity and virulence analyses

Selected tomato isolates (*n* = 29) representing a range of morphological characteristics of *Fusarium* species were tested for their pathogenicity and aggressiveness on tomato leaves. Isolates were grown in Potato Dextrose Broth (PDB) media for two to four days. Spore suspension was conducted by adding sterile distilled water to fully grown mycelia, and the concentration of spore suspension was measured and adjusted to 1×10^6^ conidia per ml in water using a haemocytometer. To produce tomato seedlings for inoculation, seeds of tomato cultivar ‘Rio Grande’ were planted in a 15 × 25 cm size tray. After one week of emergence, seedlings were transplanted to small 18 cm pots. Two weeks after transplanting the tomato seedlings, the experiment was setup as a complete randomized design with three replications. For each isolate three fully expanded leaves were placed on a filter paper placed on wire mesh platforms placed in 46 × 20 cm (length × width) size plastic trays. Water was added to each tray just beneath the surface of filter paper to provide sufficient humidity to the leaves. To facilitate infection, leaves were wounded with a sharp razor blade on the midrib just before inoculation. Leaves were inoculated by pouring 10 μl of spore suspension (1×10^6^ conidia per ml water) on the wounded midribs. As a negative control, 10 μl sterile distilled water was poured on wounded midribs. Trays were covered to maintain high humidity and were incubated at 28°C for seven days. Data were analysed using a one-way ANOVA and means were compared using multiple comparison test in the GraphPad Prism 7 software to identify significant differences in disease scores among the isolates.

### Morphological Identification

For morphological characterization, first single-spore cultures were isolated and grown on PDA medium as follows. To obtain single spore colonies, isolates were initially grown on PDA medium for 7 to 10 days under 10/14 hrs of light/dark conditions. Conidial suspension was prepared by adding 15 ml sterile distilled water directly to each plate and conidia were dislodged with a sterile pipette. To obtain a single germinating conidium, two drops of the suspension was poured on water agar in petri-dishes and were incubated on a shaker for 7–10 hrs at 27°C. The germinating conidia of each isolate were transferred to PDA plates and incubated for 7 days at 25–28°C with 10/14 hrs ligh/dark conditions for pure culture growth. Cultures of each isolate were examined for texture, color, margins, and pigmentation of colonies. For microscopic examination, permanent slides for each isolate were made and were examined for mycelium charcteristics, spore sizes, and types under a confocal microscope using 10X and 60X magnifications. Macrocinidia and microconidia were differentiated on the basis of length and septation, while chlamydospores were differentiated on the basis of absence or presence and intercalary or terminal position on the mycelium.

### Genomic DNA extraction

A pure culture of isolates was grown on PDB or 10% V8 medium for 48 hrs at 28°C. The mycelial pads were harvested with a sterile spatula and were stored in 1–5 mL Eppendorf tubes at -80°C for DNA extraction. Genomic DNA was extracted using the DNeasy^®^ Plant Mini Kit (cat. No. 69104, QIAGEN USA) according to the manufacturer’s protocol. The purity and concentration of the extracted DNA was determined by measuring the absorbance value at 260 and 280 nm using the Take3 Micro-Volume plate with a Synergy H1 Hybrid Multi-Mode Reader (BioTek.com). DNA samples were stored at -20°C and -80°C as working and stock solutions for further use.

### ITS sequencing

Internal Transcribed Spacer region (ITS) of the rDNA gene was amplified using primers ITS1 (5′-TCCGTAGGTGAACCTGCGG-3′), ITS4 (5′-TCCTCCGCTTATTGATATGC-3′) and ITS5 (5′-GGAAGTAAAAGTCGTAACAAGG-3′) [29]. For ITS1 and ITS4, the amplification reactions were performed in a total volume of 10 μl consisting of DNA (0.5–5 ng), dNTP (0.2 mM each), each primer (0.3 μM), Taq DNA polymerase (0.625 U), 1× PCR buffer and 2 mM MgCl_2_. For ITS1 and ITS5 amplification, reactions were performed in a total volume of 20 μl consisting of 10 μl 2× HiFi PCR mix, 4.5 μl of 2 μM each primer and 0.5–5 ng DNA. PCR amplification conditions consisted of an initial denaturation step at 98°C for 2 min followed by 35 cycles of denaturation at 98°C for 10s, annealing at 55°C for 30 s and extension at 72°C for 90s, and a final extension at 72°C of 10 minutes. All PCR experiments included a negative control (distilled water without DNA) and a positive control consisting of DNA of a known *F*. *o*. f.sp. *lycopersici* isolate (Fl-15).

The ITS-PCR reactions were run on a 1% agarose gel containing SYBER green dye in 1× TAE buffer at 80 volts for 40 minutes. Gels were visualized and photographed with Gel DOC XR software (Universal Hood II, USA). After electrophoresis, single approximately 500 bp long bands from each lane were isolated using the Qiagen QIA quick Gel Extraction Kit (cat#28704) according to the manufacturer's recommended protocol. The concentration of the PCRed ITS DNA was quantified using Take3 Micro-volume plate with a Synergy H1 Hybrid Multi-Mode Reader (BioTek.com). The ITS DNA was sequenced on both strands using ITS1, ITS4 and ITS5 primers using Sanger sequencing. DNA chromatogram files of all sequences were checked manually using the Geneious R8 software. For each isolate, ITS sequences of both strands were assembled into consensus contigs using the Geneious R8 software. The consensus contigs were then searched at the FUSARIUM-ID [30] and the NCBI nucleotide databases using BLASTn, the closest BLAST match was identified based on percent coverage, and percent nucleotide identity and E-values.

### *TEF-1α* sequencing

A portion of the translation-elongation factor -1 alpha gene (*TEF-1α*) from all 30 *Fusarium* isolates was amplified using primer pairs EF1 (5′-ATGGGTAAGGARGACAAGAC-3′) and EF2 (5′-GGARGTACCAGTSATCATGTT-3′). PCR amplification was conducted using the TaKaRa Taq^TM^ polymerase kit (Cat R001A) in a 50 μl reaction volume containing template DNA (5 ng), dNTPs (0.2 mM each), primers EF1 and EF2 (0.3 μM each), Taq DNA polymerase (0.625 U), and 1× PCR buffer with final 1.5 mM MgCl_2_. PCR amplification conditions consisted of an initial denaturation step at 98°C for 2 min, followed by 35 cycles of denaturation at 98°C for 10s, annealing at 55°C for 30 s, and extension at 72°C for 90s, and a final extension at 72°C of 10 minutes. PCR reactions were run on a 1% agarose gel containing SYBER green dye in 1× TAE buffer at 80 volts for 40 minutes. Gels were visualized and photographed with the Gel DOC XR software (Universal Hood II, USA). After electrophoresis, DNA bands from each lane were isolated using the Qiagen QIA quick Gel Extraction Kit (cat#28704). The amplified DNA was sequenced using EF1 and EF2 primers using Sanger sequencing. DNA chromatograms were manually examined in the Geneious R8 software. Sequences of both strands were assembled into contigs for each isolate and the consensus contigs were then searched at the *FUSARIUM-ID* v 1.0 [30] and the NCBI nucleotide databases using BLASTn.

### Phylogenetic analyses

Both ITS and *TEF-1α* sequence data sets were aligned using Multiple Alignment using Fast Fourier Transform (MAFFT)[31]. Phylogenetic analyses were conducted using the Unweighted Pair Group Method with Arithmetic Mean (UPGMA) algorithm using the Tamura-Nei genetic distance model [32] using the Geneious R8 software. Consensus dendrograms and bootstrap values for both data sets were inferred from 1,000 replicates. The trees were rooted with *Fusarium oxysporum* f.sp. *lycopersici* as type species.

### Inter-simple sequence repeats analysis

Two ISSR primers, (GA)_9_C and (GA)_9_T, were selected for investigating genetic relationships among all the 30 *Fusarium* species isolates. The ISSR-PCR amplification was carried out using the TaKaRa Taq^TM^ Kit (Cat R001A) in a 20 μl reaction volume containing template DNA (5 ng), dNTP (0.2 mM each), an ISSR primer (0.6 μM), Taq DNA polymerase (0.625 U) and 1× PCR buffer with 2 mM MgCl_2_. Sterile distilled water used as a negative control. The PCR program consisted of an initial denaturation at 98°C for 30 seconds, followed by 35 cycles of denaturation at 94°C for 1 min, annealing at 45°C for 1 min, extension at 72°C for 2 min and a final extension at 72°C for 10 min. PCR amplification was performed in a Gene Amp PCR System 9700 Thermocycler (PE Applied Biosystems). The amplified PCR products were run on 1% agarose gel stained with SYBR green dye in 1× TAE buffer and run for 40 minutes at 80 volts. The gene ruler 1kb plus DNA ladder (0.5 μg/μl) was used as a marker (Cat#SM1331, Thermo Scientific USA). The ISSR analysis (DNA extraction, PCR, and electrophoresis) was repeated three times to confirm the reproducibility of the primers for each isolate. Gels were visualized, and gel images were captured with Gel DOC XR and compared with 1kb DNA ladder as a marker (Universal Hood II, USA).

The amplified DNA fragments from ISSR analysis were converted to binary data matrix in excel sheet (0 = no band, 1 = presence of the band). To check the genetic relatedness among and within different *Fusarium* species, the data matrix of the ISSR markers was converted into distance matrix using Jaccard's coefficient in R version 3.0.3(https://cran.r-project.org/). The resulting distances were used to construct a phylogenetic tree using the UPGMA method. Bootstrap analysis for genetic distance search with 1,000 replicates was performed to obtain the confidence of cluster analysis. *Fusarium* species population structure parameters including the number of polymorphic bands, the percentage of polymorphic bands, observed number of alleles (N_A_), effective number of allele (N_E_), Nei’s genetic diversity (H), Shannon’s information index (I_S_), total genetic diversity (H_T_), genetic diversity within groups (H_S_) and relative magnitude of genetic differentiation between populations (GST = (H_T_-H_S_)/H_T_, gene flow (N_m_), genetic identity (I), and genetic distance (D) were analysed using POPGENE version 1.3.1 [33] [34].

## Results

### Disease incidence, pathogenicity analyses and morphological identification

**Fig 1:**
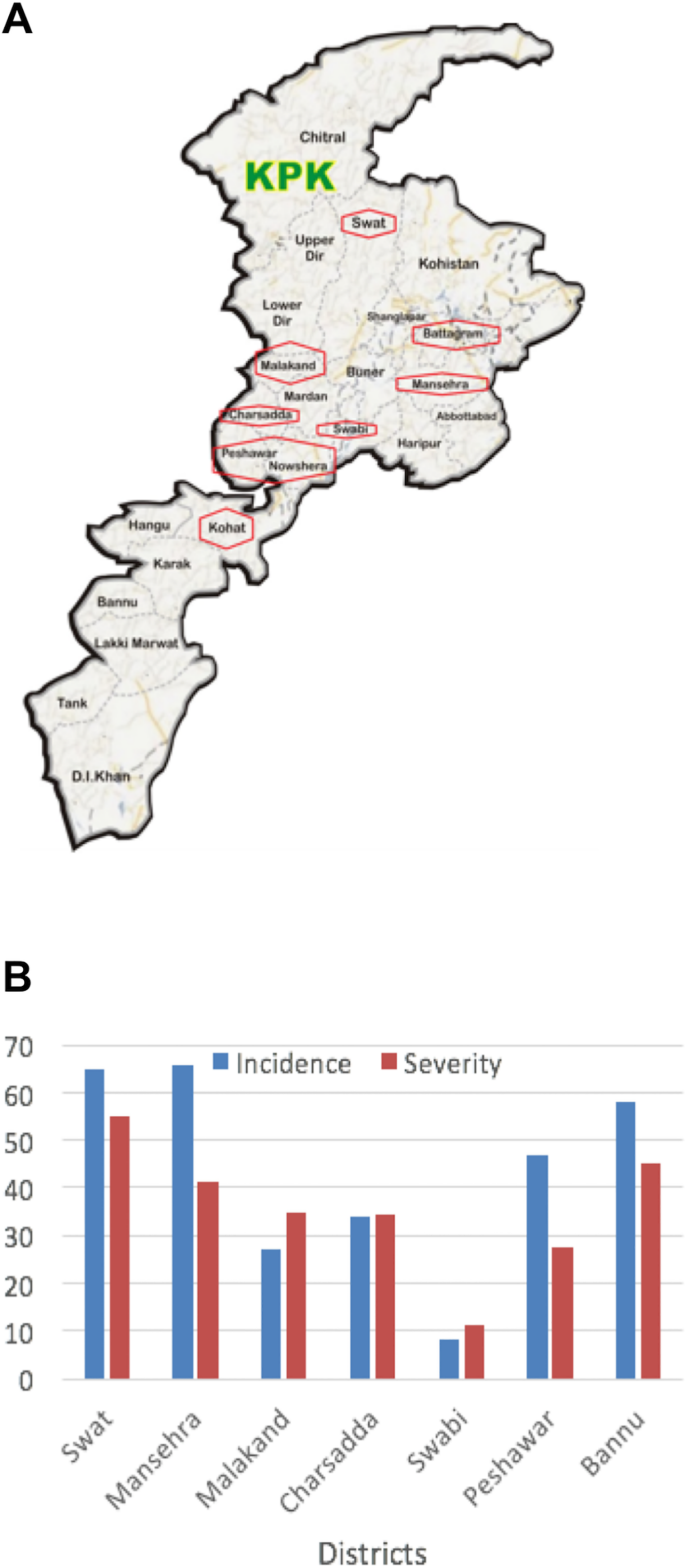
Geographical distribution of *Fusarium* species in Northern Pakistan. **(A)** Map of Khyber Pakhtunkhwa showing locations of surveyed areas **(B)** Bar graph showing incidence and severity of Fusarium wilt in tomatoes in Khyber-Pakhtunkhwa.

According to growers, disease incidence is generally increasing every year, perhaps due to inadequate control measures and inappropriate and/or inaccurate fungicide rates and application methods. Visual observations of the surveyed tomato fields revealed presence of characteristic *Fusarium* symptoms such as wilting, vascular discoloration, and yellowing of leaves at different growth stages ranging from young seedlings to mature flower- and fruit-bearing plants throughout the crop season. In general, incidence and severity of the disease were higher in Northern (Swat and Mansehra) and Southern (Peshawar and Bannu) districts, and comparatively lower in central districts (Malakand, Charsadda, and Swabi) (Fig 1).

**Fig 2:**
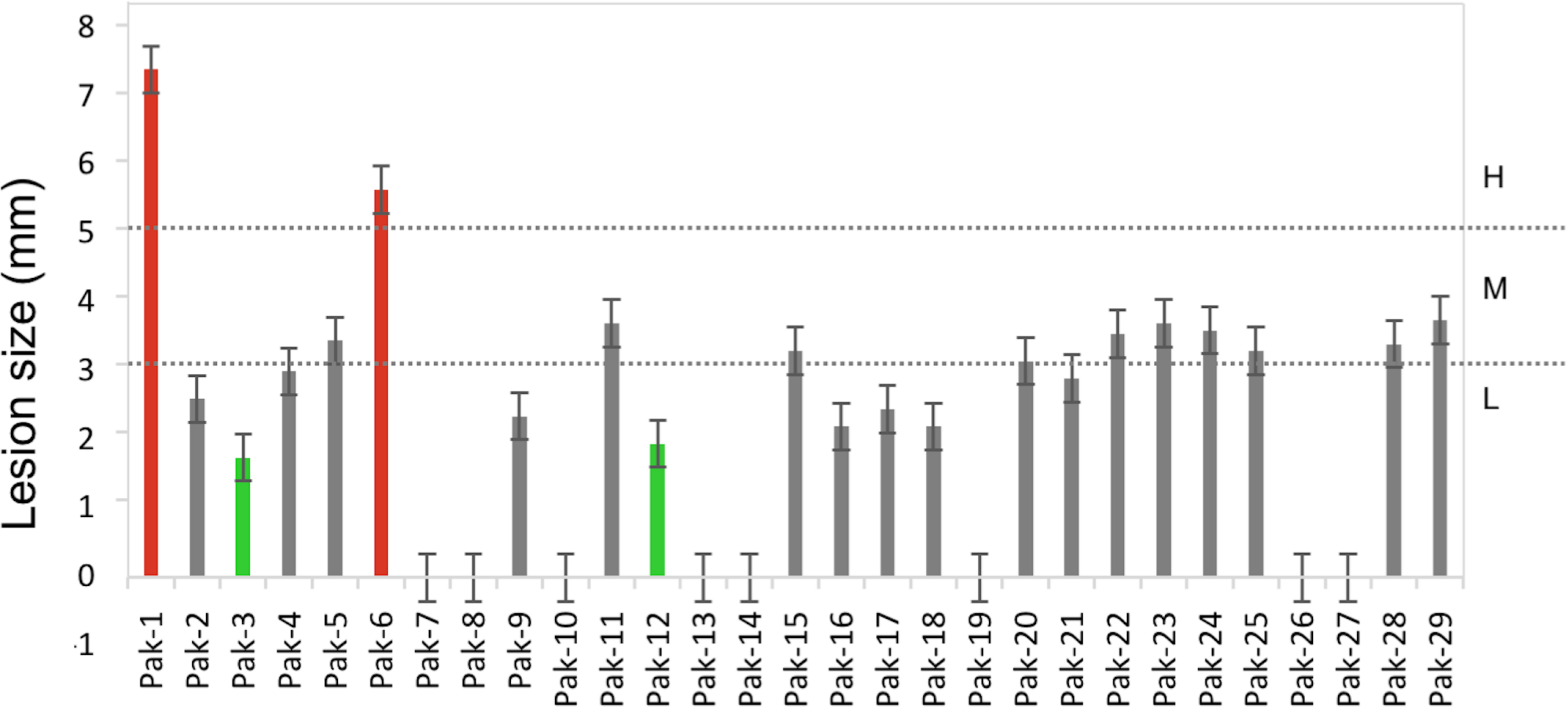
Pathogenicity of *Fusarium* isolates based on results of detached tomato leaf assays. Data are the average lesion size ±SD of three replications. Dotted lines demarcate boundaries among High (H, lesion size > 5 mm), Moderate (M, lesion size 3–5 mm) and Low (L, lesion size < 3 mm) virulence of isolates.

Pathogenicity tests conducted using a detached leaf inoculation method with *Fusarium* species isolates showed differential responses (Table 1 and Fig 2). Based on lesions sizes, isolates were classified as highly virulent (H, lesion size > 5 mm), moderately virulent (M, lesions size 3–5 mm), and less virulent (L, lesion size < 3 mm). Multiple comparison tests of the lesion size data showed no significant difference among most isolates except Pak-1 and Pak-6, which were classified as highly virulent with lesion sizes of 6 and 7 mm, respectively (Fig 2). Isolates Pak- 4, Pak-5, Pak-11, Pak-15, Pak-20, Pak-21, Pak-22, Pak-23, Pak-24, Pak-25, Pak-28, and Pak-29 belonged to the moderately (M) virulent class. Isolates Pak-2, Pak-3, Pak-9, Pak-12, Pak-16, Pak-17, and Pak-18 were classified as less (L) virulent isolates. Isolates Pak-10, Pak-14, Pak-19, Pak-27, Pak-7, Pak-8, Pak-13, and Pak-26 were non-pathogenic.

Macroscopic and microscopic characteristics of all the isolates, including colony characteristics, mean spore length of macro and microconidia, presence and absence of chlamydospores are summarized in Table 1. Based on morphological characteristics, all isolates were tentatively classified into four different *Fusarium* species groups (Table 1**)**. The identified species included *Fusarium solani* (Pak-21, Pak-29), *Fusarium equiseti* (Pak-1, Pak-2, Pak-3, Pak-4, Pak-5, Pak-6, Pak-9, Pak-11, Pak-12, Pak-15, Pak-16, Pak-17, Pak-18, Pak-20, Pak-22, Pak-23, Pak-24, Pak-25, Pak-28, Pak-29), *Fusarium graminearumm* (Pak-10, Pak-14, Pak-19, Pak-27) and unknown *Fusarium* species (Pak-7, Pak-8, Pak-13, Pak-26).

### Identification based on ITS and *TEF-1α* gene analyses

All 30 isolates were identified using DNA sequences of *ITS* and *TEF-1α*. Targeted regions of both *ITS* and *TEF-1a* regions were successfully amplified from all isolates. BLASTn analyses conducted using nucleotide databases at the *FUSARIUM-ID* [30] and NCBI (http://www.ncbi.nlm.nih.gov/) revealed a 98–100% identity for *ITS* and 99–100% identity for *TEF-1a* with the reported *Fusarium* species isolates. Based on ITS sequences, 19 isolates were identified as *F*. *equiseti*, four as *F*. *graminearum*, three as *F*. *acuminatum*, two as *F*. *solani*, and one as *F*. *verticillioides* (Table 1). BLASTn analyses of the *TEF-1a* sequences accurately classified all but two isolates, Pak-13 and Pak-26, similar to classification based on *ITS* (Table 1). A majority of the isolates were identified as *F*. *equiseti*, which belongs to the *Fusarium incarnatum-equiseti* species complex (FIESC) (Table 1). Two isolates that were identified as *F*. *verticillioides* (Pak-13) and *F*. *acuminatum* (Pak-26) based on ITS sequences were identified as *F*. *graminearum*. Differences between the results of a few isolates based on morphology, and ITS and *TEF-1a* sequencing are most likely due to close resemblance in morphological and genetic characteristics among many closely-related species in the genus Fusarium. Similarly, ITS sequences are not always accurate in accurately classifying Fusarium species [11]. Since, analysis based only on ITS sequencing can result in misidentifying a species, classification based on other genes such as *TEF-1a* is recommended [11]. As is shown in Table 1, based on the morphological, and ITS and *TEF-1a* sequencing, of the total 29 isolates tested, 20 isolates (68.9%) belonged to FIESC, 6 isolates (20.7%) belonged to *F*. *graminearum*, 2 isolates (6.8%) belonged to *F*. *acuminatum* and 2 isolates (6.8%) belonged to *F*. *solani*.

### Phylogenetic inference

**Fig 3:**
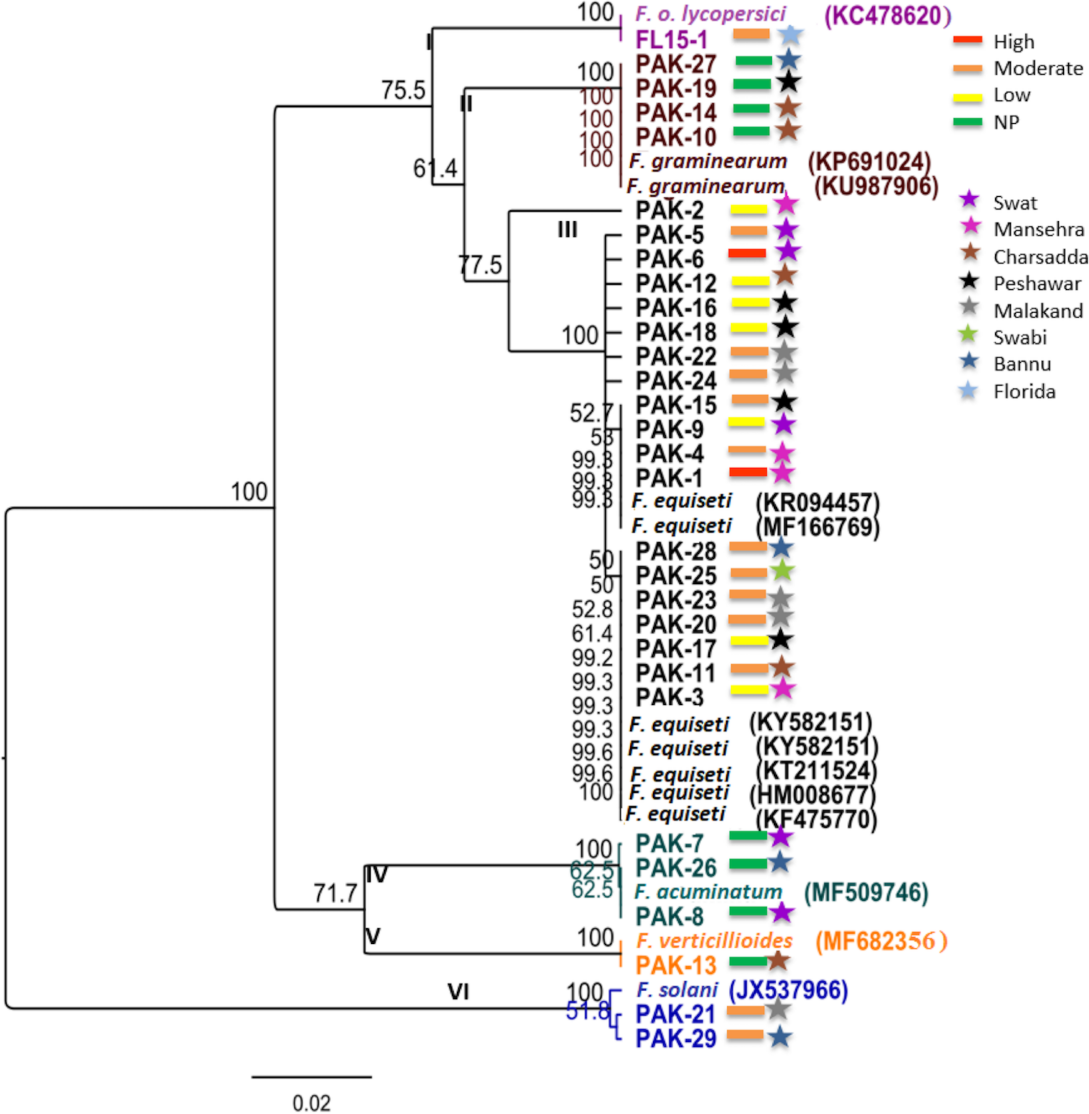
Phylogenetic analysis of *Fusarium* species isolates based on rDNA ITS sequences using the UPGMA method. The tree includes reference strains of *F*. *o*. f.sp. *lycopersici* and of other species identified. Bootstrap support was calculated from 1000 replicates. NP stands for non-pathogenic. Color of bars represent virulence levels, and color of stars represent different geographic locations.

Phylogenetic analysis of the ITS sequences classified all isolates into six major groups with high bootstrap values (Fig 3). Sixty five percent isolates belonged to clade III, which contained heterogeneous isolates of *F*. *equiseti*. Isolates in clade III represented different geographical areas and different levels of virulence including the most virulent isolates, Pak-1 and Pak-6. One pathogenic isolate, Pak-2, with low virulence from district Swat formed a separate branch within clade III. The *F*. *graminearum* isolates (Pak-10, Pak-14, Pak-19, and Pak-27) formed a separate clade (Clade II) supported by 100% bootstrap values. Isolates in this group were non-pathogenic on tomatoes. Interestingly all isolates in this group were from the Southern part of the province. Clade IV and V consisted of non-pathogenic isolates, which were identified as *F*. *acuminatum* and *F*. *verticillioides*. Clade VI consists of only *F*. *solani* isolates.

**Fig 4:**
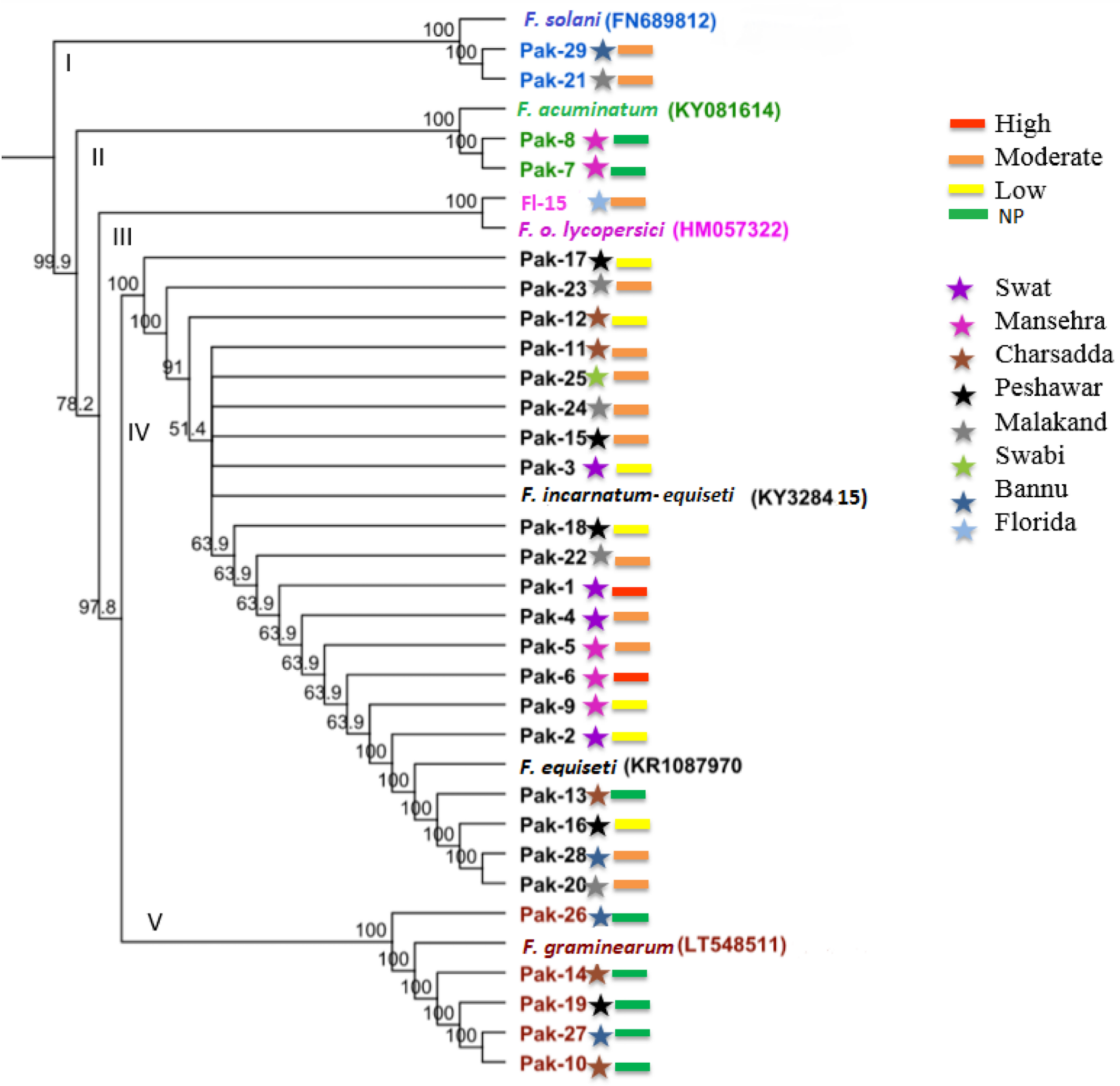
Phylogenetic analysis of *Fusarium* species isolates based on *TEF-1α* sequences using the UPGMA method. The tree includes reference strains of *F*. *o*. f.sp. *lycopersici* and of other species identified. Bootstrap support was calculated from 1000 replicates. NP stands for non-pathogenic. Colors of bars represent virulence levels, and colors of stars represent different geographic locations.

The phylogenetic tree constructed based on the *TEF-1α* gene separated *Fusarium* population into five major clades, which were all supported by high bootstrap values (Fig 4). Clade IV contained the largest number of isolates, which all belonged to *F*. *equiseti*. Clade II and Clade V mainly consisted of non-pathogenic isolates. Except Pak-13, Clade IV consisted of all pathogenic isolates irrespective of geographic distribution. Phylogenetic tree based on *TEF-1α* sequences efficiently separated subclades and identified isolates to the subspecies level.

### Genetic diversity based on ISSR analyses

**Fig 5:**
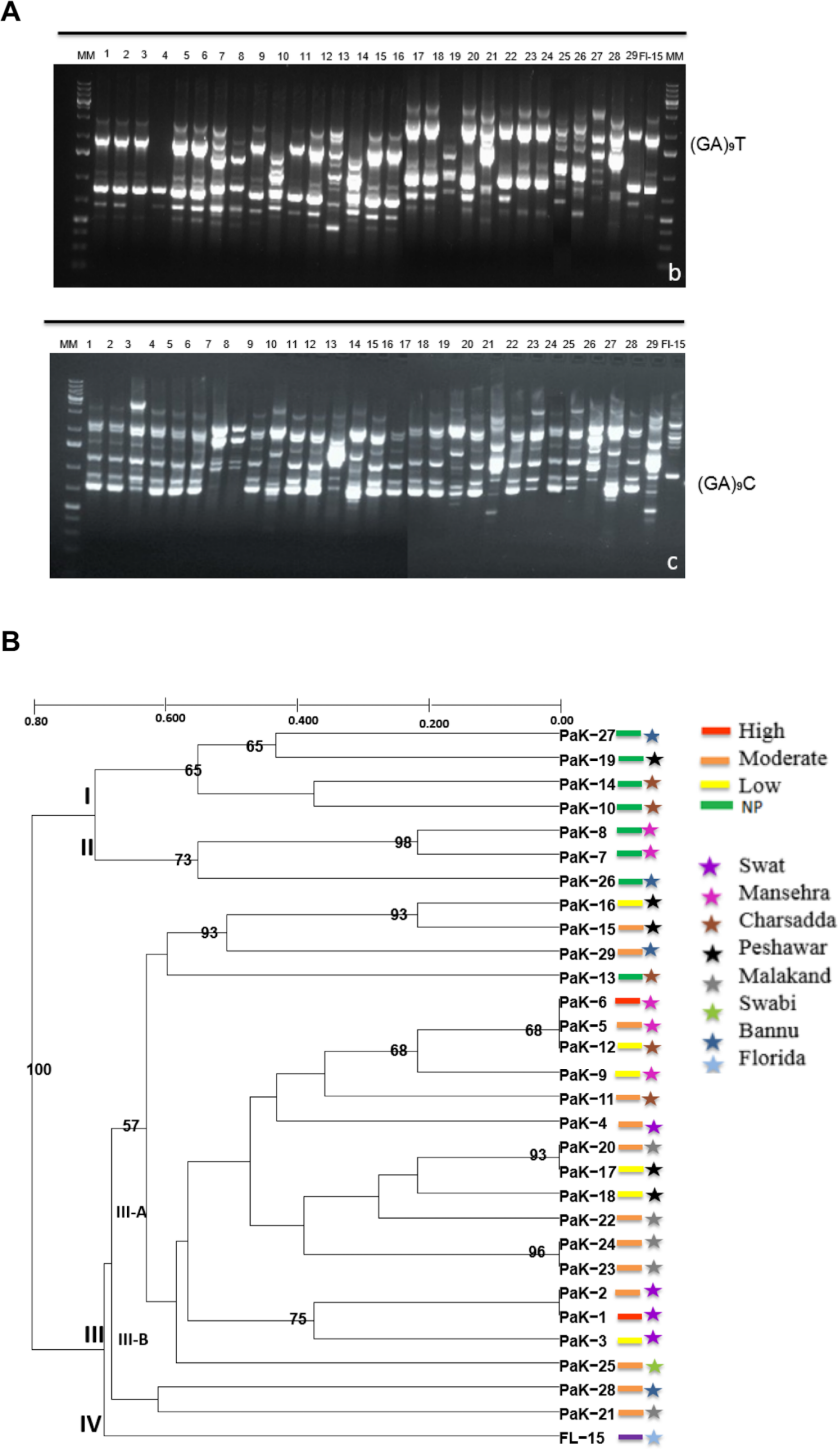
(**A)** Genetic fingerprints based on ISSR-PCR analyses with the ISSR primer (GA)_9_T and (GA)_9_C of 30 *Fusarium* species isolates. **(B)** UPGMA dendrogram of *Fusarium* species isolates based on the ISSR data showing relationship among different isolates reported in this study. Values at the nodes indicate confidence intervals of 1000 replicates. NP stands for non-pathogenic. Bootstrap values less than 50% were omitted. Colors of bars represent virulence levels, and colors of stars represent different geographic locations.

The degree of genetic diversity among and within different *Fusarium* species associated with wilt of tomato was evaluated using ISSR analyses. The ISSR primers (GA)_9_T and (GA)_9_C showed robust results and they were selected based on their high retention power and reproducibility in producing polymorphic band patterns for all the 30 isolates representing five different species of *Fusarium*. These primers produced a total of 303 bands ranging in size from 400–4500 bp. However, the minimum and the maximum number of polymorphic ISSR fragments varied from three to seven per isolate (Fig 5A).

Based on ISSR fingerprinting, genetic diversity among different species of *Fusarium* isolates from tomato was assessed by calculating the distance matrix, which ranged from 0.00 to 0.80 (Fig 5B). Using these genetic distances, a UPGMA dendrogram was constructed, which classified all isolates into four major clades. Clade I and Clade II consisted of non-pathogenic isolates of *F*. *graminearum* and *F*. *acuminatum* irrespective of the geographic distribution of the isolates. Interestingly, Clade III consisted of all pathogenic isolates, including highly virulent, moderately virulent, and low virulent isolates except Pak-13. Isolates Pak-1, Pak-2, Pak-5, Pak-6, Pak-12, Pak-17, and Pak-20 all had a genetic distance of 0.00, showing that these isolates are closely related. Clade III was subdivided into two sub-clades, III-A and III-B. Clade IV consisted of the single type strain *F*.*o*. f.sp. *lycopersici* from Florida as an outgroup (Fig 5B).

Genetic diversity parameters of different populations based on geographic origin are shown in Table 2. Among the four populations- North, Mid, South and a reference strain from Florida—Na ranged from 1.889 to 1.00 with an average of 1.629; Ne from 1.486 to 1.00 with an average of 1.344; and H varied from 0.286 to 0.00 with an average of 0.2055. The average number of polymorphic bands were 17. Similarly, Ht of the 30 isolates was 0.307, Hs was 0.205, Gst was 0.331, and total gene flow was 1.011. Matrices of genetic identity (I) and genetic diversity (D) among the four populations were obtained from the pairwise comparison **(**Table 3). Results showed that highest genetic identity was between the populations from the South and central regions (0.9706). The highest genetic distance was between the population from the central region and the reference strain *F*. *o* f.sp. *lycopersici* from Florida (0.3662), while the lowest distance was between the South and central regions (0.0298).

**Table 2:** Genetic diversity and gene flow of *Fusarium* species in Khyber-Pakhtunkhwa.

Regions	PB^a^	PPB^a^ (%)	N_A_^a^	N_E_^a^	H^a^	I^a^	H_T_^a^	H_S_^a^	G_ST_^a^	N_M_^a^
**North**	24	88.89	1.889	1.486	0.286	0.434	-	-	-	-
**Central**	19	70.37	1.704	1.426	0.252	0.377	-	-	-	-
**South**	25	92.59	1.926	1.465	0.284	0.438	-	-	-	-
**Florida**	0	0	1.000	1.000	0.000	0.000	-	-	-	-
**Average**	17	62.962	1.629	1.344	0.2055	0.312				
**Among regions**							0.307	0.205	0.331	1.011

^a^ Genetic diversity parameters used in this studies are: PB, number of polymorphic bands; PPB, percentage of polymorphic bands; N_A_, observed number of alleles; N_E_, effective number of alleles; H, Nei’s gene diversity; I, Shannon’s information index; H_T_, total genetic diversity; H_S_, Genetic diversity within groups; G_ST_, relative magnitude of genetic differentiation between populations; N_M_, gene flow.

**Table 3:** Nei’s genetic identity and genetic distance.

	North	Central	South	Florida
**North**	-	0.9611	0.9679	0.7459
**Central**	0.0396	-	0.9706	0.6934
**South**	0.0326	0.0298	-	0.7312
**Florida**	0.2932	0.3662	0.3130	-

Nei’s genetic identity (above diagonal) and genetic distance (below diagonal)

## Discussion

To the best of our knowledge, this is the first study reporting epidemiological and phylogenetic analyses of *Fusarium* species infecting tomatoes in Khyber-Pakhtunkhwa province. Our study shows that *Fusarium* wilt is present in a majority of the tomato growing areas at all growth stages, most likely because of lack of sanitation, using infected seeds, and mono-culturing, which could lead to build-up of soil-borne inoculum. In addition, inappropriate use of fungicides could also lead to the development of fungicide-resistant strains thus increasing the prevalence and incidence of the disease [35].

Incidence of the disease was higher in the northern and southern regions of the province and lower in the central region. Soil in the northern area is sandy clay loam with an acidic pH 5–6 and a temperature range of 25–28°C during tomato growth season, which is suitable for *Fusarium* infection and survival [36–38]. In district Swat, growers do not practice crop rotation and they also grow a limited number of tomato varieties, which can lead to resistance break down and selection of virulent fungal isolates. Similarly, in southern regions, growers do not practice crop rotation and they rely on a small number of varieties, which are usually grown from non-certified infected seeds from local sources. Furthermore, soil conditions in this region were dryer with very low moisture content, which can predispose plants to *Fusarium* attack as reported earlier [39]. Due to lack of extension education, farmers were unaware of the benefits of disease-free seeds and crop rotation. Since *Fusarium* is a soil- and seed-borne pathogen, seedlings are readily infected right after emergence when grown from infected seeds. It is well known that crop rotation increases disease suppressive conditions, which help reduce soil-borne inoculum [40, 41]. Thus, increasing crop rotation and introducing genetically diverse varieties could lead to improved management of *Fusarium* diseases in tomatoes. Use of healthy disease-free seeds would also reduce disease incidence and fungicide sprays. Reducing fungicide sprays in turn would help reduce emergence of fungicide-resistant virulent strains.

In contrast, in the central region of the province, disease incidence and severity were lower. In these areas, growers use Nitrate fertilizers, which, by raising soil pH, appear to limit the growth of *Fusarium* [42]. Growers in this region also reported rotating tomato crop with leguminous crops such as beans and peas, which is consistent with reports showing that crop rotation limits the incidence of *Fusarium* wilts. Furthermore, including leguminous crops in rotation allows nitrogen-fixing bacteria such as *Rhizobia* to fix atmospheric nitrogen to nitrates and nitrites, which raise soil pH that could limit the growth of *Fusarium* species and reduce disease incidence in tomatoes [40, 43].

Pathogenicity assay showed that two *Fusarium* isolates, Pak-1 and Pak-6, were highly virulent on tomato leaves whereas the remaining 27 isolates were either moderately and weakly pathogenic or non-pathogenic on tomatoes. The difference in virulence may be due to differences in genes responsible for pathogenicity and virulence and/or genetic makeup of host plants that could differentiate pathogenic isolates from non-pathogenic isolates [44].

Based on morphological identification using micro and macroconidia, *F*. *equiseti* was found to be the most prevalent *Fusarium* species followed by *F*. *graminearum*, *F*. *solani*, and *F*. *acuminatum* associated with tomatoes in Khyber-Pakhtunkhwa. Our results are supported by finding in other studies, which reported different *Fusarium* species including *F*. *oxysporum*, *F*. *verticillioides* [45], *F*. *redolens*, *F*. *proliferatum*, *F*. *equiseti*, and *F*. *solani* associated with wilt of tomatoes [46–50]. Most studies focused on diagnostic and phylogenetic analyses of *Fusarium* species use ß-tubulin, translation elongation factor (*TEF-1α*), RPB 1 and 2, intergenic spacer (IGS), mitochondrial small subunit (mtSSU) rRNA, and internal transcribed spacer (ITS) region of the ribosomal RNA genes [11, 51–54]. Identification based on morphology is not always accurate. In this study, we used ITS and *TEF-1α* genes, which identified all isolates accurately to the species levels. Our results showed that *TEF-1α* sequences outperformed ITS in identifying *Fusarium* species associated with the disease, particularly for members of the FIESC by providing superior resolution. It is also reported that for some fungi, such as *Trichoderma* and *Fusarium*, *TEF-1α* are recommended for species elucidation [13]. ITS sequences usually do not provide sufficient genetic information for accurately resolving taxonomic position of recent *Fusarium* lineages in phylogenetic trees [55]. In general, slowly evolving genes such as *TEF-1α*, and RPB 1 and 2 are more reliable for inferring deep phylogenies, whereas gene sequences undergoing faster evolutionary rates such as TUB2 are more susitable for recent evolutionary and speciation events. The *TEF-1α* gene satisfy both these requirements consisting of both conserved exonic and variable intronic sequences [55]. According to result of this and other studies, it is important to take into consideration that ITS sequencing alone is not sufficient for correct identification of *Fusarium* species associated with a disease.

Based on molecular identification, *Fusarium equiseti*, which belongs to *F*.*incarnatum-equiesti* species complex (FIESC), was the main species responsible for wilt of tomato in Khyber-Pakhtunkhwa. Within the FIESC clade, isolates from different areas were scattered with no evidence of phylogenetic structure with respect to their pathogenicity and geographic origin, which are consistent with previous findings [56]. The second most common *Fusarium* species pathogenic on tomato plants in Khyber-Pakhtunkhwa was *F*. *solani*, which displayed moderate virulence. *F*. *solani* is a common pathogen of tomato causing wilt, and pre- and post-emergence damping off in different parts of the world such as India and Turkey [57, 58]. In Israel, *F*. *solani* is the principal cause of wilt of tomato in *F*. *oxysporum* resistant tomato varieties [59].

*Fusarium* is a complex genus with many similar, different, and not well-researched species. To sustain durable resistant or tolerant varieties, which is the most efficient mean of control for *Fusarium* wilt of tomato, knowing changes in the genetic diversity of different *Fusarium* species pathogenic on tomato is imperative [60]. Employing ISSR markers for genetic analysis are very efficient as ISSR is a simple, robust and a low-cost PCR-based method, which precisely measures genetic differences both at the genus and species level compared to other markers such as RAPD and AFLP [60]. ISSR analysis of the twenty nine *Fusarium* isolates, identified in our study as *F*.*equiesti*, *F*. *graminearum*, *F*. *solani*, *F*. *acuminatum*, and the type species *F*. *o*. f.sp. *lycopersici*, revealed high genetic diversity among and within populations of *Fusarium* species isolated from tomato in Khyber-Pakhtunkhwa. Cluster analysis showed that pathogenic and non-pathogenic isolates of different *Fusarium* species tend to cluster separately irrespective of their geographic distribution, indicating that there is some correlation between the pathogenic and non-pathogenic isolates. These results are consistent with a previous study showing that genetic diffferentiation and genetic distances are not associated with geographic distribution [19]. Furthermore, this could be explained by the spread of identical genotypes in different geographical areas by long-distance distribution of infected seeds or planting materials. High genetic diversity within the *F*. *equiesti* and *F*. *solani* isolates reflected polyphyletic nature of *Fusarium* species usually attributed to high evolutionary rate within the microsatellite regions [61]. However, some isolates of the *F*.*equiesti* were closely related and showed low genetic diversity. This narrow variation could be due to agricultural practices in which growers mainly rely on local varieties thus restricting the introduction of new genotypes of the pathogen [62]. Similar studies have been conducted on other *Fusarium* species of several major crops showing that ISSR markers are a reliable and authentic source for inferring genetic relationship within and between different *Fusarium* species [24, 60].

In this studies, we report the genetic structure of *Fusarium* isolates based on parameters such as percentage of polymorphism (PP), observed number of alleles (N_A_), effective number of alleles (N_E_), Nei’s gene diversity (H), Shannon’s information index (Is), and N_m_ gene flow [63]. We reported highest genetic diversity (0.286) in *Fusarium* population from the Norther region of the province. Given the fact that a majority (68.9%) of these isolates belonged to *F*. *equiseti* indicated high intraspecific variation within the *F*. *equiseti* isolates. The high genetic variation among different *Fusarium* species and the genetic partitioning within *F*. *equiseti* are in agreement with the estimates of expected heterozygosity reported for other *Fusarium* species [24, 64]. In phylogentic studies, a gene flow (N_m_) value of 1.0 and a genetic differetiation (G_ST_) value of 0.25 are considered as treshholds, beyond which significant differentiation occurs [65]. Considereing these principles, *Fusarium* populations reported in our studies were just above the treshold level (N_m_ = 1.011, G_ST_ = 0.33). We reported the highest genetic identity (0.9706) and lowest genetic distance (0.0298) among the poulations of southern and central regions, which could be due to local tranfer of planting material among growers of these areas [66].
